# Modeling HIV-1 Drug Resistance as Episodic Directional Selection

**DOI:** 10.1371/journal.pcbi.1002507

**Published:** 2012-05-10

**Authors:** Ben Murrell, Tulio de Oliveira, Chris Seebregts, Sergei L. Kosakovsky Pond, Konrad Scheffler, on behalf of the Southern African Treatment and Resistance Network (SATuRN) Consortium

**Affiliations:** 1 Biomedical Informatics Research Division, eHealth Research and Innovation Platform, Medical Research Council, Tygerberg, South Africa; 2 Computer Science Division, Department of Mathematical Sciences, Stellenbosch University, Stellenbosch, South Africa; 3 Africa Centre for Health and Population Studies, Nelson R. Mandela School of Medicine, University of KwaZulu-Natal, Durban, South Africa; 4 Research Department of Infection and Population Health, University College London, London, United Kingdom; 5 School of Computer Science, University of KwaZulu-Natal, Durban, South Africa; 6 Department of Medicine, University of California San Diego, San Diego, California, United States of America; Imperial College London, United Kingdom

## Abstract

The evolution of substitutions conferring drug resistance to HIV-1 is both episodic, occurring when patients are on antiretroviral therapy, and strongly directional, with site-specific resistant residues increasing in frequency over time. While methods exist to detect episodic diversifying selection and continuous directional selection, no evolutionary model combining these two properties has been proposed. We present two models of episodic directional selection (MEDS and EDEPS) which allow the *a priori* specification of lineages expected to have undergone directional selection. The models infer the sites and target residues that were likely subject to directional selection, using either codon or protein sequences. Compared to its null model of episodic diversifying selection, MEDS provides a superior fit to most sites known to be involved in drug resistance, and neither one test for episodic diversifying selection nor another for constant directional selection are able to detect as many true positives as MEDS and EDEPS while maintaining acceptable levels of false positives. This suggests that episodic directional selection is a better description of the process driving the evolution of drug resistance.

## Author Summary

When exposed to treatment, HIV-1 and other rapidly evolving viruses have the capacity to acquire drug resistance mutations (DRAMs), which limit the efficacy of antivirals. There are a number of experimentally well characterized HIV-1 DRAMs, but many mutations whose roles are not fully understood have also been reported. In this manuscript we construct evolutionary models that identify the locations and targets of mutations conferring resistance to antiretrovirals from viral sequences sampled from treated and untreated individuals. While the evolution of drug resistance is a classic example of natural selection, existing analyses fail to detect the majority of DRAMs. We show that, in order to identify resistance mutations from sequence data, it is necessary to recognize that in this case natural selection is both episodic (it only operates when the virus is exposed to the drugs) and directional (only mutations to a particular amino-acid confer resistance while allowing the virus to continue replicating). The new class of models that allow for the episodic and directional nature of adaptive evolution performs very well at recovering known DRAMs, can be useful at identifying unknown resistance-associated mutations, and is generally applicable to a variety of biological scenarios where similar selective forces are at play.

## Introduction

Among positively selected evolutionary changes, a distinction can be made between *diversifying selection*, where any nucleotide substitutions that change the amino acid are favored, and *directional selection*, where only substitutions towards a small number of target amino acids are selected for. Detection of genes or sites evolving under positive selection [1]–[6] has been dominated by methods which explicitly or implicitly assume *diversifying* positive selection. This assumption allows evolution to be modeled as a continuous-time Markov process without assuming that any particular residue is the preferred target of substitutions at any sites. For most models of diversifying selection, apart from a single rate governing amino acid change, the process is no different from one site to the next. By contrast, models have been proposed in which specific residues do have special status at specific sites. In models of toggling selection [7], substitutions away from a site-specific “wild type” amino acid are likely to be followed by reversions to that amino acid. Models of directional selection [8], [9] allow substitution rates towards a site-specific “target” amino acid to be accelerated. By making a distinction among all possible targets of a substitution, such models allow the detection of positive selection favoring mutations towards one amino acid, even at sites where the overall rate of amino acid change is decreased by purifying selection. For a review of codon models of selection, see [10].

A second distinction is that between selective pressure that is constant over time, and selective pressure that changes over time, possibly instantaneously – we shall refer to the latter as *episodic selection*. Several authors have studied models that allow evolutionary rates to change over time, including models in which the selective pressure is different on different branches [11]–[14] as well as various models [15]–[17] in which the rate of evolution at any site may change at any point in time. We are specifically interested in the former type of model, under which rate changes occur simultaneously at a particular set of sites - as would be expected under an external change in selective pressure, *i.e.* episodic selection. This type of selection is applicable to countless real world scenarios that have been studied extensively: examples include the evolution of lysozyme in response to diet changes [18], the adaptation of HIV to different host populations [14], the evolution of the rhodopsin pigment following changes in habitat [19], and the adaptation of HIV-1 [20], [21] and Influenza A Virus (IAV) [22] genes following zoonosis events. For a review on the evidence for episodic selection in large numbers of protein sequences, see [23].

Here, we consider the evolution of drug resistance in HIV-1 following the treatment of a subset of the host population. We expect that selective pressure will be both episodic, with drug-induced adaptive amino acid changes occurring only in patients receiving therapy, and directional, with site-specific target residues increasing in frequency over time in the treated subset. HIV-1 experiences a variety of other selective pressures, most prominently due to host immune response (e.g. [14], [24]), but because such response is nearly unique in each host, we expect that the majority of concerted selective changes in subjects on treatment will be drug-induced.

Previous approaches to detect positive selection driving treatment resistance have had variable success. Crandall *et al.* [25] showed that normalized ratios of non-synonymous to synonymous substitution counts (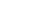) obtained by the counting method of Nei and Gojobori [1] failed to show consistent evidence of selection, despite obvious resistance associated substitutions occurring in parallel in many patients. Chen *et al.* [26] used a contingency-table counting method to characterize positive selection towards specific amino acids in a sample of approximately 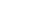 sequences. However, their approach ignored the phylogenetic relationships between samples which can cause selection to be conflated with founder effects [22], [27]. Lemey *et al.* [28] used the branch-site model of Yang and Nielsen [12] – a model of episodic diversifying selection – to analyze the evolution of drug resistance over a transmission chain. A number of sites were inferred to be under positive selection, of which some were associated with drug resistance. Seoighe *et al.* [8] modeled the evolution of reverse transcriptase between pre- and post-treatment samples from 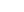 patients. They successfully detected some of the major drug resistance mutations with few false positives.

In this paper we aim to demonstrate that explicitly modeling the directional and episodic character of the evolution of drug resistance increases the power and accuracy to detect drug resistance sites. We introduce a codon-based Model of Episodic Directional Selection (MEDS) and a model of protein evolution called Episodic Directional Evolution of Protein Sequences (EDEPS), and show that both models outperform models that lack either the episodic or directional components.

## Models

### MEDS

Our codon model of episodic directional selection assumes that branches on the phylogenetic tree can be partitioned into foreground (F) and background (B) subsets *a priori*. Evolution along background branches is described by a standard codon model (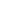, see below). In the model for foreground branches (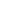), directional selection is incorporated via an elevated rate of substitutions towards a target amino acid.

MEDS extends two previously proposed models of coding sequence evolution: 1) the episodic component of MEDS is structurally identical to the Internal Fixed Effects Likelihood (IFEL) model proposed in [14], although IFEL is used to detect diversifying selection along internal branches only, and, 2) the directional component is introduced in a manner similar to that in the model of directional selection proposed by Seoighe *et al.* [8]. We used 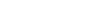 [29] as our baseline codon model: it combines a general time-reversible (GTR) model of nucleotide substitution with separate synonymous (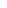) and non-synonymous (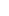) rates. To facilitate reading, table 1 summarizes the properties of each model.

**Table 1:** Summary of models described in this manuscript.

Model	Data	Baseline model	Site variation	Lineage variation	Selection test	Citation
MEDS	Codon	MG94 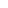 REV^a^	Fixed effects	Episodic	Directional	This paper
FEEDS	Codon	MG94 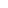 REV	Fixed effects	Episodic	Diversifying	[14] b
DEPS	Protein	HIV-Between^c^	Random effects	Constant	Directional	[9]
EDEPS	Protein	HIV-Between	Random effects	Episodic	Directional	This paper

a [29].

b FEEDS has the same structure as a model called IFEL in that paper, but the use here is novel.

c [37].

Following Seoighe *et al.* [8] we add a directional selection parameter 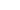 to modulate the rate of substitutions to the target residue 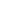 in the model assigned to foreground branches. If 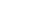 represents the amino-acid encoded by codon 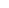, then the instantaneous rates of change between codons 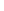 and 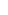 (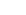) are given by:
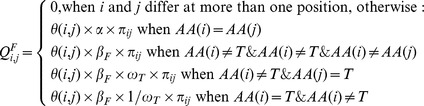
(1)for the foreground and 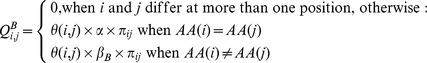
(2)for the background branches. We assume that 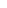 does not change significantly between foreground and background branches. Indeed, available evidence (e.g. [30]–[32]) suggests that synonymous rate variation among sites is due to biological processes which change slowly, e.g. RNA secondary structure, transcriptional or translational efficiency, relative to the nearly instant change in the selective environment due to the presence of ARV. In principle, the model can readily handle such variation. 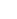 and 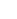 can be inferred independently. 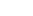 is the GTR-based rate of the underlying nucleotide substitution from codon 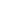 to 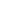, shared between 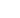 and 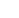. Equilibrium frequency parameters 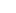 are derived with the corrected 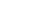 estimator [33]. While the same 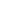 values are used for background and foreground models, when 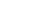 the equilibrium frequencies of 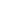 will depart from those dictated by 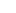, although we do not need to calculate these new equilibrium frequencies explicitly. This feature is essential because directional evolution changes the character frequencies at a site. We also experimented with a version of the model where the factor 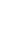 in the last line of Equation 1 was omitted – this had essentially no impact on the results. To ensure that 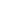 defines a valid Markov process generator, along the diagonal of 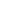
we set:
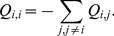
(3)


Model fitting proceeds in two stages: (a) estimating the parameters shared across sites, and (b) site-wise analysis [6], [34]. The branch lengths and 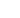 and 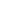, without the directional component (*i.e.* 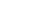), are first optimized over the entire alignment to obtain gene-wide parameter estimates in the presence of potentially ubiquitous purifying or diversifying selection. The nucleotide rate parameters (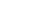) and relative branch lengths are then fixed for subsequent analyses. From then, the analysis proceeds site by site. We define the null model by setting 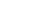, a special case of the alternative directional model (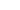 is free to vary), and equivalent to IFEL [14]. The null model has 3 free parameters per site: 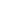 (synonymous substitution rate), 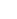 (non-synonymous substitution rate along foreground lineages) and 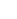 (non-synonymous substitution rate along backfround lineages). The alternative model has a single additional parameter, 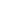, biasing substitutions towards 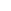. To test for selection towards amino acid 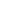 at a specific site, we obtain maximum likelihood scores for the null and alternative models and perform a likelihood-ratio test (LRT) with one degree of freedom based on the asymptotic 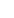 distribution of the likelihood-ratio statistic.

The above test treats nucleotide substitution rates and branch length parameters at a single site as known, even though these are estimated across sites under a simpler model. It is possible that this could affect inference if these estimates were substantially biased. Our simulations suggest that the test performs well in spite of this computational shortcut, and using different models to infer these parameters does not substantially affect the test results on the empirical data we analyze here. Additionally, the 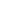 asymptotic approximation implicit in MEDS relies on the intuition that when the number of sequences increases, the number of branches in the tree will increase, so that substitutions on those branches will constitute different (although dependent) realizations of the process. We note that the asymptotic approximation for our test requires not only many branches but also many foreground branches. While theoretical results justifying our use of the 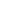 approximation are currently lacking, our simulations (see below) suggest that the use of the 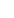 appears to lead to a conservative test for the conditions we have examined.

Scanning a site for selection towards any possible amino acid (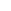) involves testing 20 hypotheses, and we employ Bonferroni correction [35] to control the site-wise Type I error rate. For computational efficiency, we skip invariant sites and restrict potential values of 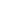 to those observed at a given site. Because these reductions are informed by the data, we still employ the 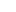-test Bonferroni correction at each site.

### FEEDS

To assess the importance of the directional component of MEDS, we adapt IFEL to test for episodic diversifying selection along foreground branches and use it as a benchmark for MEDS. As the branches of interest are mostly terminal, the name, IFEL, is no longer appropriate, and we rename the model FEEDS, for ‘Fixed Effects Episodic Diversifying Selection’. The alternative model for FEEDS is identical to the null model for MEDS, allowing 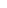, 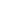 and 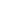 to vary for each site. To test for non-neutral selection along foreground branches, we set up a null model with 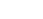, and use an LRT (one degree of freedom) to determine whether the alternative model fits better than the null model. If 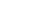 results in a significant likelihood improvement, we have evidence for diversifying selection along foreground branches. This test for episodic diversifying selection has three features that distinguish it from the popular branch-site model of Yang and Nielsen [12] and Zhang, Nielsen and Yang [36]: 1) it uses a sitewise likelihood-ratio test [5], otherwise known as a fixed effects likelihood [6] approach, 2) it allows site-to-site synonymous rate variation, which has been shown to be ubiquitous and can cause spurious detection of diversifying selection if ignored [29] and 3) it allows diversifying selection on the background branches in both the null and alternative models. MEDS shares these properties, allowing us to attribute any performance differences specifically to the directional component of MEDS.

### DEPS

Throughout the analyses we also compare our results against DEPS (full results in tables S1 to S3), a method for detecting non-episodic directional selection proposed by Kosakovsky Pond *et al.* [9]. DEPS identifies sites with increased substitution rates towards specific amino acids, but it differs from MEDS in three ways: 1) DEPS models directional selection at the amino acid level rather than the codon level, 2) DEPS uses a Random Effects Likelihood (REL) framework to bias selection towards target amino acids across all sites, relying on an empirical Bayes analysis to identify sites of interest and 3) in DEPS, directional selection affects all branches of the phylogeny.

### Episodic DEPS

It is a straightforward exercise to modify DEPS to incorporate the episodic nature of MEDS – namely, we restrict accelerated substitutions towards a target residue 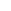 (and retard substitutions away from it) to foreground branches, while background branches always evolve according to the baseline protein substitution model specific to HIV-1 [37]. The entire testing framework of DEPS, as described in Kosakovsky Pond *et al.* [9], applies without change. It is well known that amino acid substitution rates depend on the residues involved (e.g. see [38]), and specifying a baseline model which includes unequal substitution rates provides a qualitative advance over MEDS. Conversely, because DEPS works with protein sequences, the natural proxy of approximately neutral evolution (the rate of synonymous substitutions) is not available.

All models and their accompanying LRTs are implemented in a HyPhy Batch Language script [39], and all code and test datasets are available on the MEDS section of the HyPhy wiki (www.hyphy.org) and included in the latest HyPhy distribution (version 2.0020101225 or later).

### Datasets

We analyzed three HIV-1 datasets obtained from the South African mirror of the Stanford HIV Drug Resistance Database (HIVdb) [40], [41]. Synthetic datasets were generated by simulation to investigate the power and false positive rate of MEDS. The primary goal of this paper is to show that MEDS and EDEPS perform well on medium-sized datasets constructed under a variety of conditions. Every empirical dataset includes sequences sampled from both treated and untreated patients, but we varied the inclusion criteria from one dataset to the next. An ideal dataset for detecting drug resistance would include pre- and post-treatment samples from the same patients (as in our reverse transcriptase dataset), but often such data are not available, e.g. when sequences are obtained from patients experiencing regimen failure. To evaluate the performance of MEDS and EDEPS when pre- and post-treatment sequence pairs were not available (our protease and integrate datasets), we selected pre-treatment sequences using heuristic measures of proximity to the post-treatment samples, as one would be forced to do under such circumstances. Exactly which factors are responsible for performance variation is left as a topic for future research. The objective of each analysis was to detect sites (and corresponding amino acids) that are involved in drug resistance. For validation, we used the curated list of drug resistance associated mutations (DRAMs) which is available from the Stanford HIVdb (http://hivdb.stanford.edu). This list is produced every year and approved by the International AIDS Society (http://www.iasusa.org/resistance_mutations/). These mutations have been rigorously validated with genotype-phenotype and genotype-clinical data and are known to confer varying levels of resistance to one or more antiretroviral agents – they can therefore be used as a ground truth for evaluating the performance of our methods.

We screened each sequence for evidence of recombination (known to have a biasing effect on selection detection, e.g. [42]) using SCUEAL [43] and excluded any sequences showing 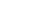 support for either inter- or intra-subtype recombination, and using the Rega HIV-1 Subtyping tool Version 2.0 [44], excluding any sequences with clear inter-subtype recombination.

#### Reverse transcriptase

The first dataset comprises pairs of reverse transcriptase (RT) isolates obtained before and after the initiation of highly active anti-retroviral therapy (HAART) from 241 patients (482 sequences). The data were obtained from the Stanford HIVdb using a query that retrieved paired samples from the same patient, filtered on the earlier sample being Reverse Transcriptase Inhibitor (RTI) naive, and the later sample taken during therapy with at least one Non-Nucleoside RTI (NNRTI) *and* at least one Nucleoside RTI (NRTI). The topology of the phylogeny was estimated using PhyML [45] (settings for all datasets: REV model with tree search by Nearest Neighbor Interchange and Subtree Pruning and Regrafting), and all terminal branches leading to post-treatment sequences were selected as foreground (see Figure S1). As an artifact of older sequencing assays [14], a large number of sequences were missing data at the beginning and end of RT, hence we analyzed the region from codon 40 to 250. Six sequences were excluded from our analyses because they displayed evidence of recombination.

#### Protease

**Figure 1:**
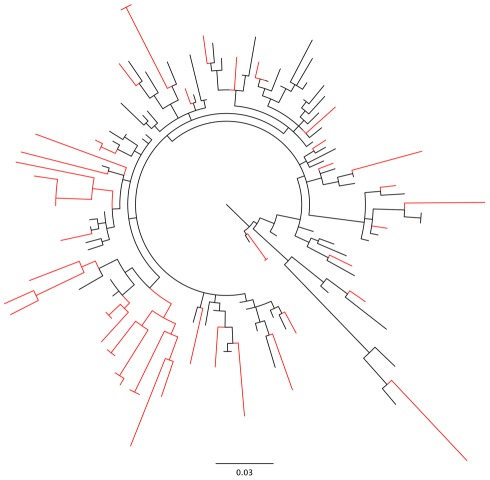
The maximum-likelihood phylogeny for the protease dataset. Foreground branches are marked in red. All terminal foreground branches lead to sequences obtained from patients who had been receiving antiretroviral therapy. See text for details of how we determined which internal branches were assigned to foreground. MEDS and EDEPS allow the presence of a directional component along the foreground branches where antiretroviral therapy exerts selective pressure.

A dataset consisting of 49 protease isolates (from 37 patients), sampled post-Protease Inhibitor (PI) treatment was retrieved from HIVdb (query: Number of PIs = 3, Subtype = C). Additionally, the entire collection of treatment naive protease isolates was obtained, and all full length sequences were searched for two sequences nearest (under the Hamming distance) to each of the 49 post-treatment sequences. The final dataset was constructed by combining the post-treatment and closely related naive sequences: a total of 122 sequences, as some naive sequences were closely related to more than one post-PI sequence. Since protease is only 297 nucleotides long, we were concerned that convergent evolution due to drug resistance might inflate the apparent relatedness between some of the treatment resistant sequences [46], hence we excluded the major resistance sites before reconstructing the phylogeny, using PhyML. As there are many instances where a number of post-treatment sequences were sampled from a single patient, we adopted a recursive branch labeling strategy for the internal branches. All terminal branches leading to post-PI and PI-naive isolates were labeled as foreground and background respectively, and internal branches were labeled as foreground if both child branches were foreground, and background otherwise (See figure 1). This labeling ensures that drug resistance selection occurs only on foreground branches. Because there may be portions of foreground branches not under drug selection, the effect of potential mislabeling is to dilute the signal along foreground branches and reduce the power of the test. No sequences showed evidence of recombination.

#### Integrase

The post-treatment sequences for the final empirical dataset were 83 integrase isolates sampled from 40 patients after Integrase Inhibitor (II, Raltegravir) therapy. 1237 II-naive isolates were obtained from the Stanford HIVdb, and the final Raltegravir dataset was made up of 315 sequences: the 83 post-II isolates, plus the union of the 25 II-naive isolates nearest to each of the 83 post-II isolates under the HKY85 distance [47]. The topology of the phylogeny was again estimated using PhyML, and the foreground region was labeled in the same fashion as the protease dataset (see Figure S2). 20 sequences were excluded for showing evidence of recombination.

#### Power simulations

We investigated the power of MEDS by simulating alignments over a balanced 64-taxon phylogeny (see Figure S3 for an example). All parameters were varied (see Text S1 for complete details). Of particular interest, we simulated under 4, 8, 16 or 32 foreground branches and, selecting a random target amino acid 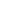 for each site, the directional selection parameter 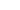 took values of 2, 5, 10, 100 and 1000. These 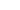 values are in a reasonable range: in our three empirical datasets, the 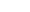, 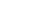 and 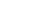 percentiles of the maximum-likelihood estimates of 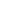 values for detected substitutions are 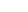, 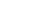 and 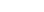. 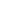 sites were simulated for each 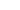 value, for each number of foreground branches, yielding 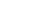 simulated sites. To assist in understanding the effects of 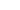 and the size of the foreground subset, we also recorded the number of substitutions towards the target amino acid that occurred along foreground branches.

In real evolving systems, the modeling assumption of selection towards a single target amino acid could be violated. We investigated how such deviations would impact the power of the model by simulating directional selection towards two target amino acids, with substitutions towards one target accelerated on 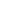 foreground branches, and substitutions towards another accelerated on a different 8 foreground branches. The parameters were varied in the same manner as the single-target power simulation, and 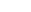 sites were simulated for each 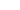 value, again yielding 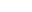 sites in total.

#### False positive simulations

We used exactly the same simulation configuration and parameters to asses the rates of false positives under the null model (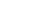). We simulated 400 sites for each of 4, 8, 16 or 32 foreground branches.

In evolving proteins, each site could have its own site-specific selective constraints governing amino acid distributions. MEDS assumes that background equilibrium frequencies are the same for all sites, and a potential concern is that deviations from this modeling assumption could lead to excessive false positives. To investigate this, we simulated data under a version of the null model where each site's amino acid equilibrium frequencies were sampled from a symmetric Dirichlet distribution with density
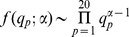
(4)The concentration parameter 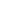 took values 0.005, 0.05, 0.5 and 5, varying the equilibrium frequency distributions from extremely peaked to relatively flat. Each sampled amino-acid frequency 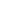 was evenly distributed among all codons encoding 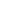 and a version of 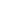 with the Goldman-Yang parameterization of equilibrium frequencies [4] was employed to simulate codon sequence data.

## Results

### Reverse transcriptase

MEDS detected twenty substitutions at seventeen sites under significant directional selection at 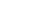, after correcting for multiple tests (see tables 2 and S4). Of these, five are known NRTI drug resistance associated mutations (DRAMs) (41L, 116Y, 151M, 184V and 215F) and six are known NNRTI DRAMs (100I, 103N, 181I, 188L, 190S and 230L). Additionally, 228R is listed as an accessory NRTI mutation. The eight detected substitutions that have not been experimentally or clinically associated with drug resistance are 64K, 98S, 104Y, 151Q, 165L, 188Y, 215T and 286A. Interestingly, at three of these sites (151, 188 and 215) selection was detected both towards the wildtype and towards resistant residues. EDEPS agreed with MEDS on eleven sites, detected additional DRAMs 62V, 77L and 115F, missed two MEDS-reported DRAMs (41L and 116Y), and found episodic selection at 162S and 174R which are not known to confer drug resistance.

**Table 2:** Sites under episodic directional and episodic diversifying selection in reverse transcriptase.

Site	Target	MEDS p-value^a^	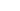 (Lower 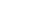 CI)^b^	FEEDS p-value^c^	EDEPS Bayes Factor^d^	Resistance
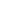	L	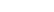	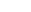 (280.06)	-e	-	NRTI^f^
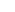	V	-	-	-	313	NRTI
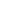	K	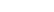	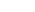 (5.58)	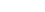	-	
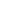	L	-	-	-	211	NRTI
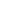	S	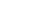	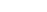 ( 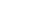 )	-	-	
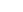	I	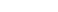	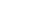 (524.49)	-	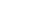	NNRTI^g^
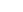	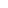 h	-	-	0.0025	-	
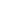	N	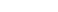	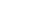 (466.73)	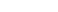	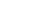	NNRTI
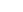	Y	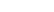	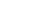 (90.81)	-	-	
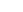	F	-	-	-	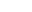	NRTI
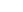	Y	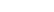	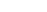 (179.80)	-	-	NRTI
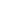	M	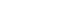	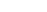 (186.13)	-	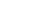	NRTI
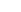	Q	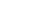	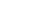 (7.04)	-	-	
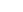	S	-	-	-	1772	
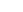	L	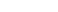	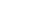 ( 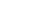 )	-	2245	
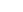	R	-	-	-	105	
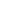	I	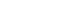	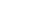 (118.72)	-	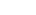	NNRTI
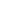	V	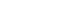	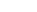 (16.68)	-	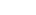	NRTI
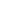	L	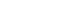	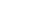 (32.42)	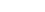	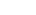	NNRTI
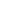	Y	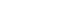	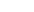 (11.15)	-	-	
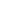	S	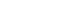	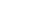 (26.09)	-	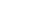	NNRTI
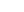	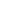	-	-	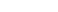	-	
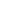	F	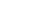	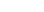 (10.36)	-	2727	NRTI
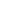	T	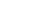	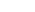 (6.69)	-	-	
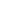	R	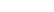	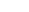 (14.09)	-	1401	NRTI accessory
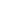	L	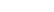	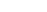 (44.6)	-	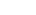	NNRTI
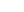	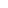	-	-	0.0006	-	
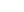	A	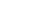	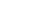 ( 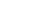 )	-	-	

a MEDS versus FEEDS LRT, testing for directional selection.

b the lower bound of the approximate 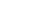 confidence interval calculated from profile likelihood.

c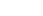 LRT, testing for diversifying selection.

d Empirical Bayes analysis, testing for directional selection on protein data.

e ‘-’: not significant.

f Nucleoside reverse-transcriptase inhibitor.

g Non-nucleoside reverse-transcriptase inhibitor.

h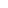: detected only by FEEDS which does not identify a target AA.

Remarkably, FEEDS detected only six sites under diversifying selection (table S5), two of which are known resistance mutations, strongly supporting the inclusion of a directional component in the model. A continuous directional selection model (DEPS) detected 46 sites under directional selection with Bayes factors 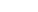 (see table S1), only ten of which are on the HIVdb list. This indicates that focusing our attention on branches where the evolutionary environment shifts is advantageous for finding evidence of adaptive response to such shifts.

### Protease

MEDS detected nine substitutions under directional selection at 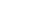 (tables 3 and S6). Of these, two are major DRAMs (90M and 84V). Three are accessory polymorphic mutations (13V, 60E and 93L) under selective pressure from the drugs. 74S is a non-polymorphic accessory mutation. EDEPS agreed with MEDS on three (13V, 84V and 90M), detected one more major mutation, 82A, and an accessory mutation at 71V. Interestingly, 60E and 61E found by MEDS involve substitutions (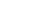 and 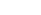) which, in HIV, are much more frequent than the mean substitution rate [37]. Because MEDS sets the background rate of non-synonymous substitutions to the same value for all pairs of residues, it could use 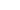 to compensate for the *overall* underestimation of rates that are much greater than the mean rate.

**Table 3:** Sites under episodic directional and episodic diversifying selection in protease.

Site	Target	MEDS p-value^a^	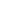 (Lower 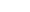 CI)^b^	FEEDS p-value^c^	EDEPS Bayes Factor^d^	Resistance
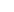	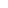 e	-f	-	0.0005	-	PI^g^ accessory
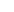	T	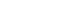	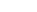 (8.58)	-	-	
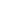	V	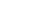	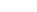 (138)	-	145	PI accessory
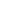	D	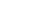	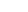 (1.99)	-	-	
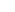	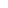	-	-	0.0026	-	PI
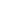	E	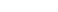	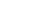 ( 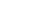 )	-	-	PI accessory
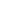	E	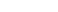	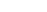 ( 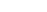 )	-	-	
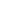	V	-	-	0.0011	257	PI accessory
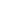	S	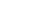	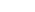 (4.08)	0.0013	-	PI accessory
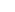	A	-	-	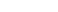	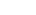	PI
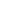	V	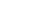	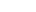 (248.19)	-	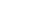	PI
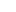	M	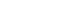	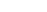 (986.17)	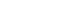	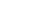	PI
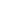	L	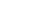	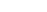 (6.36)	-	-	PI accessory

a MEDS versus FEEDS LRT, testing for directional selection.

b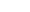 lower confidence interval calculated from the likelihood profile.

c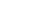 LRT, testing for diversifying selection.

d Empirical Bayes analysis, testing for directional selection on protein data.

e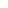: detected only by FEEDS which does not identify a target AA.

f ‘-’: not significant.

g Protease inhibitor.

FEEDS identified six sites involved in diversifying selection (table S7), with all six listed on HIVdb. In addition to two sites already detected by MEDS (74 and 90), sites 10 and 71 are listed as accessory mutations, while 54 and 82 are major resistance mutations. DEPS appeared to be much more conservative on this dataset, detecting four sites under directional selection, two of which are listed on HIVdb (see table S2).

### Integrase

MEDS detected six substitutions under significant directional selection at the 1% level (see tables 4 and S8). Four (140S, 143R, 148H and 155H) appear on the HIVdb list of mutations associated with a 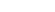 fold decrease in Raltegravir susceptibility. Two are listed as mutations selected by Raltegravir (72I and 97A). EDEPS confirmed five DRAMs (97A, 140S, 143R, 148H and 155H), together with a 163R accessory substitution and a 221Q mutation which is not a known DRAM.

**Table 4:** Sites under episodic directional and episodic diversifying selection in integrase.

Site	Target	MEDS p-value^a^	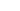 (Lower 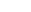 CI)^b^	FEEDS p-value^c^	EDEPS Bayes Factor^d^	Resistance
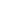	I	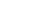	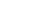 (533.76)	-e	-	INI^f^ accessory
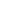	A	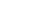	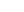 (105.52)	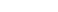	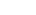	INI accessory
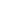	S	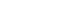	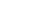 ( 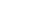 )	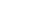	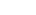	INI
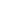	R	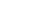	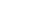 (3.83)	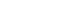	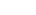	INI
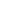	H	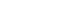	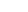 (14.53)	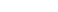	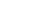	INI
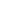	H	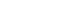	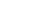 ( 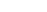 )	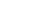	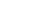	INI
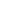	R	-	-	-	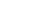	INI accessory
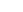	Q	-	-	-	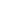	
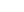	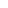 g	-	-	0.0064	-	
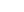	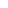	-	-	0.0048	-	INI accessory

a MEDS versus FEEDS LRT, testing for directional selection.

b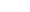 lower confidence interval calculated from the likelihood profile.

c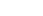 LRT, testing for diversifying selection.

d Empirical Bayes analysis, testing for directional selection on protein data.

e ‘-’: not significant.

f Integrase inhibitor.

g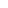: detected only by FEEDS which does not identify a target AA.

FEEDS found seven sites under diversifying selection (table S9), six of which are known resistance mutations. 230 is the only correctly identified resistance site in the integrase dataset that is detected as being under diversifying selection by FEEDS, but not directional selection by MEDS. 230 R and N are listed as selected by Raltegravir. DEPS detected 39 substitutions under directional selection (see table S3), nine of which appear on the HIVdb list.

### Comparing methods

Comparing the fit of FEEDS and MEDS on *known* resistance sites in all three datasets, LRTs reject a null model of FEEDS in favor of MEDS on 24 sites, with FEEDS being favored on five (four from protease and one from integrase). Note that FEEDS might still be useful for detecting these sites, but the LRT demonstrates that MEDS is a better model of the process. This suggests that episodic directional selection is, in most cases, a better characterization of the evolution of drug resistance. Overall, FEEDS detects fourteen true positives, while MEDS and EDEPS detect 24 each (although not the same 24). Where FEEDS appears to have a reasonably low rate of false positives but misses a large number of true positives, DEPS detects a large number of true positives but with a very high false positive rate. This is expected as DEPS will detect substitutions under selection along background branches that are not related to drug resistance.

### Power simulations

The power of MEDS, like that of other codon methods, strongly depends on the information content of the sequences, specifically on the number of times that substitutions toward the target occur along the foreground lineages. For example, even when 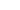 is 1000, no substitutions towards 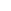 occur on half the sites simulated on the phylogeny with sixteen foreground branches. The primary reason for this is that 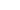 affects only the instantaneous substitution rate from a codon to its direct neighbors; if none of the direct neighbors of 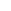 are visited along a foreground branch, a change in 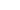 will not affect the process.

Hence, we tabulate MEDS results only for sites with at least one substitution towards the target on any foreground branch. Table 5 shows that the power is positively correlated with 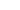. MEDS appears to be quite powerful, even when the number of foreground branches is small, achieving, for example, 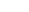 power with 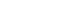 with only eight foreground branches. Table 6 displays the power of MEDS conditioned on the number of substitutions towards the target on foreground branches. With only one substitution there is almost no power, but moderate power (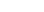) occurs with two substitutions towards 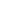, and with five or more substitutions towards 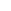, the power is almost 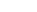.

**Table 5:** Single target power simulations: power as a function of 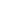.

# FG branches	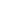				
	2	5	10	100	1000
4	0 (8)^a^	0 (16)	0 (37)	0.31 (110)	0.79 (155)
8	0 (11)	0 (18)	0.04 (62)	0.51 (129)	0.73 (170)
16	0 (31)	0.018 (54)	0.036 (83)	0.59 (177)	0.71 (201)
32	0.02 (62)	0.03 (71)	0.16 (116)	0.68 (223)	0.80 (282)

a Numbers in brackets are the number of times at least one substitution towards the target occurred along foreground branches: *i.e.* the denominator for the proportion of detections.

**Table 6:** Single target power simulations: power as a function of number of substitutions to target AA along foreground branches, pooling over 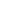.

# FG branches	# substitutions to target AA					
	0	1	2	3	4	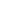
4	0 (1674)^a^	0 (119)	0.2 (58)	0.77 (48)	0.99 (111)	N/A
8	0 (1610)	0 (146)	0.23 (53)	0.69 (26)	1 (21)	0.99 (144)
16	0 (1454)	0 (200)	0.34 (92)	0.49 (39)	0.79 (34)	0.97 (181)
32	0 (1246)	0.03 (234)	0.4 (107)	0.41 (70)	0.70 (46)	0.97 (297)

a Numbers in brackets are the number of times that many substitutions towards the target occurred along foreground branches: *i.e. the denominator for the proportion of detections*.

For data simulated with two target residues, each on eight foreground branches, the occurrence of at least one substitution towards *both* targets is infrequent. From 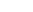 sites simulated with 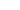 values of 2, 5 and 10, this occurs only 58 times, and is never detected. From 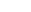 sites simulated with 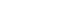 for both targets, substitutions to both targets occur 174 times. MEDS detects substitutions to at least one target in 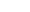 of such sites, but only detects substitutions to both targets in 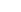 of such sites. With 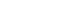, we see 306 of 1600 sites with substitutions to both targets, and MEDS detects substitutions to at least one target in 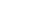 of these sites, and to both targets in 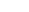.

Table 7 shows how the power increases with the number of substitutions towards both targets on the foreground branches. Since there too many possible combinations and too few observations, we display power in a cumulative manner (*i.e.* 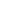 substitutions towards both targets).

**Table 7:** Dual target power simulations: power as a function of number of substitutions to two target AAs.

Substitutions to both targets^a^:	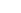	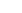	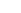	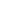	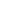	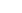	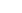	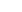
MEDS detects at least one target:	0.64	0.81	0.89	0.92	0.95	0.98	1	1
MEDS detects both targets:	0.19	0.36	0.48	0.52	0.63	0.76	0.78	0.81
Total sites:	538	288	214	179	132	99	69	32

a Substitutions along foreground branches. Each target has 8 foreground branches along which changes towards it were accelerated.

### False positive simulations

MEDS behaves conservatively. With data simulated under the null model, far fewer sites are identified as under episodic directional selection than would be expected from the nominal p-value thresholds. Across all four foreground configurations, only one false positive detection (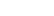, with Bonferroni correction) occurs on the 32 foreground branch phylogeny, and none on the others. With 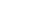, with 4, 8, 16 and 32 branches, we have false positive rates of 0, 0.0025, 0.0075 and 0.01; with 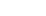, we have 0.005, 0.005, 0.0125 and 0.02, respectively. This is most likely due to FEL methods being generally conservative [6] as well as the conservative nature of Bonferroni correction. The effect of the correction is compounded because increasing the frequency of one amino acid reduces the frequency of the others, and thus the twenty tests are not independent. Table 8 shows the false positive rate for alignments simulated under site specific equilibrium frequencies. MEDS is still conservative under this scenario, and the false positive rates do not appear to be influenced by the concentration parameter.

**Table 8:** False positives with site specific equilibrium frequencies as a function of the concentration parameter 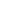 and the nominal p-value of the test.

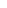 parameter:	0.005	0.05	0.5	5
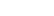	0.005	0.0025	0.0025	0.0075
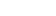	0.02	0.0175	0.02	0.015
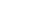	0.0325	0.0325	0.035	0.0375

## Discussion

We have proposed a codon (MEDS) and a protein (EDEPS) model of episodic directional selection, and demonstrated their performance on three HIV-1 datasets, where drug-induced directional episodic selection is expected to operate. We have also proposed a model of episodic diversifying selection (FEEDS), to rigorously evaluate the importance of modeling the directional component of natural selection. As expected, on all datasets, our episodic directional tests strongly outperform a test for continuous directional selection (DEPS) for detecting drug resistance sites. The assumptions of DEPS are inappropriate for the analysis of episodic selection, where selection is limited to specific regions of the phylogeny, because DEPS assumes uniform selection over the whole phylogeny. This serves as a caution against using suboptimal models, rather than a criticism of DEPS.

We tested MEDS with extensive simulations. MEDS is a conservative test, even when strong constraints on the amino acid state space are introduced in the form of site-specific equilibrium frequencies. Under the alternative model, good power is achieved even when relatively few substitutions towards target amino acids take place along foreground branches. When we deviate from the alternative model and elevate the substitution rate towards several target residues, the power to detect both targets is lower than it would be assuming independence. This reduction in power is expected: as the number of targets along foreground branches increases, the directional nature of the process is lost.

Hughes [48] argues that diversifying selection is only appropriate for modeling pathogen-host co-evolution, and that the constantly shifting environment is required for the standard diversifying selection model to be appropriate. Our results highlight that models of diversifying selection also serve as reasonable approximations in instances where selective constraints allow escape to many different residues, such as codon 54 in protease, which has V, T, A, L and M as major drug resistant residues. However, at most sites conferring drug resistance, directional models better approximate reality – positive selection acts only on one or a few specific mutations, while the rest are suppressed by purifying selection. The simulations presented in Table 7 illustrate how much power MEDS can be expected to have in cases such as site 54 in protease. This example also suggests a future extension of MEDS, where instead of considering one target residue at a time, substitution rates could be elevated towards *classes* of target residues.

Another interesting property of directional models is exemplified by a substitution in the protease dataset. 93L is a polymorphic mutation selected for by protease inhibitors. Despite L already being the most common residue in subtype C, the model detects selective pressure towards it – the proportion of L residues is indeed lower in nave sequences. At the population level this appears as purifying selection: the most common amino acid increases in frequency. This is nevertheless detected by our test. Far from being problematic, such information could be useful for directing treatment, if it turns out that patients with I at position 93 are more susceptible to PI therapy. Such observations should, of course, be directly verified with clinical data.

There are clear differences in organism-wide amino acid exchangeabilities in HIV-1 [37], yet the null model of MEDS (and the vast majority of other codon-models) posit that the non-synonymous substitution rate does not depend on the residues. We evaluated the effect of this assumption by comparing MEDS with an episodic version of DEPS – a test that specifically incorporates a heterogeneous exchangeability matrix in the evolutionary model. With a few exceptions, MEDS and EDEPS return overlapping sets of directionally evolving residues and identify the same targets. There are several sites in protease and integrase, where MEDS may be misclassifying non-uniform exchangeabilities as directional selection, hence another extension of MEDS would be to incorporate multiple non-synonymous substitution rates [38].

MEDS and EDEPS were designed with HIV-1 drug resistance in mind, but should be applicable wherever episodic directional selection occurs along multiple lineages. To use the models, two specific conditions must be met: 1) Lineages expected to be under directional selection must be known *a priori*, at least approximately. This is necessary to partition the phylogeny into foreground and background regions. 2) A rich collection of background sequences are needed. With HIV-1, this translates to requiring treatment naive sequences. Variety in these sequences is also important. If all the background sequences were so closely related that the foreground and background regions were separated by a single branch, if would be difficult to separate directional selection from founder effects, which would result in a loss of power. If the background sequences are spread about the phylogeny, however, founder effects are rendered unlikely and the test for directional selection should be well powered.

With HIV-1 drug resistance datasets, the foreground labeling strategy might prove important. On the RT dataset, branch-labeling was straightforward, as we had access to pre-treatment sequences for each patient. This is not the case for most real-world datasets, and other approximate labeling schemes, as well as the robustness of the results to these differences, should be investigated.

Another consideration is the rooting of the tree. With directional models, the expected amino acid frequencies change across the phylogeny, and the position of the root becomes important [9]. With MEDS and EDEPS, the directional component only affects foreground branches. Consequently, the tree can be rooted on any background branch and the likelihood will be unaffected [49].

Amidst growing concerns about an epidemic of circulating drug resistant HIV-1, the WHO and SATuRN are recommending a scale-up of drug resistance surveillance [41], [50]. This is to ensure the long-term success of the world's largest antiretroviral treatment programs, located in Africa. We see improved models of the sequence evolution playing a role in characterizing local differences in treatment resistance patterns, perhaps driven by different treatment regimens, adherence and transmission dynamics, and possibly identifying new resistance mutations.

## Supporting Information

Figure S1 The maximum-likelihood phylogeny for the reverse transcriptase dataset. Foreground branches are marked in red. All terminal foreground branches lead to sequences obtained from patients who had been receiving antiretroviral therapy.
(PDF)

Figure S2 The maximum-likelihood phylogeny for the integrase dataset. Foreground branches are marked in red. All terminal foreground branches lead to sequences obtained from patients who had been receiving antiretroviral therapy.
(PDF)

Figure S3 A balanced phylogeny used for simulations. Foreground branches are marked in red. See Text S1 for further simulation details.
(PDF)

Table S1 Reverse transcriptase results - DEPS.
(PDF)

Table S2 Protease results - DEPS.
(PDF)

Table S3 Integrase results - DEPS.
(PDF)

Table S4 Reverse Transcriptase - MEDS: Maximum likelihood parameter values for the test for episodic directional selection.
(PDF)

Table S5 Reverse Transcriptase - FEEDS: Maximum likelihood parameter values for the test for episodic diversifying selection.
(PDF)

Table S6 Protease - MEDS: Maximum likelihood parameter values for the test for episodic directional selection.
(PDF)

Table S7 Protease - FEEDS: Maximum likelihood parameter values for the test for episodic diversifying selection.
(PDF)

Table S8 Integrase - MEDS: Maximum likelihood parameter values for the test for episodic directional selection.
(PDF)

Table S9 Integrase - FEEDS: Maximum likelihood parameter values for the test for episodic diversifying selection.
(PDF)

Text S1 Simulation details. The variation in nuisance parameters used for our simulations.
(PDF)
