# Supplementary figures and images for: Modeling HIV-1 Drug Resistance as Episodic Directional Selection
Source: PLoS Comput Biol. 2012 May 10;8(5):e1002507. doi: 10.1371/journal.pcbi.1002507 (PMC3349733; doi:10.1371/journal.pcbi.1002507)

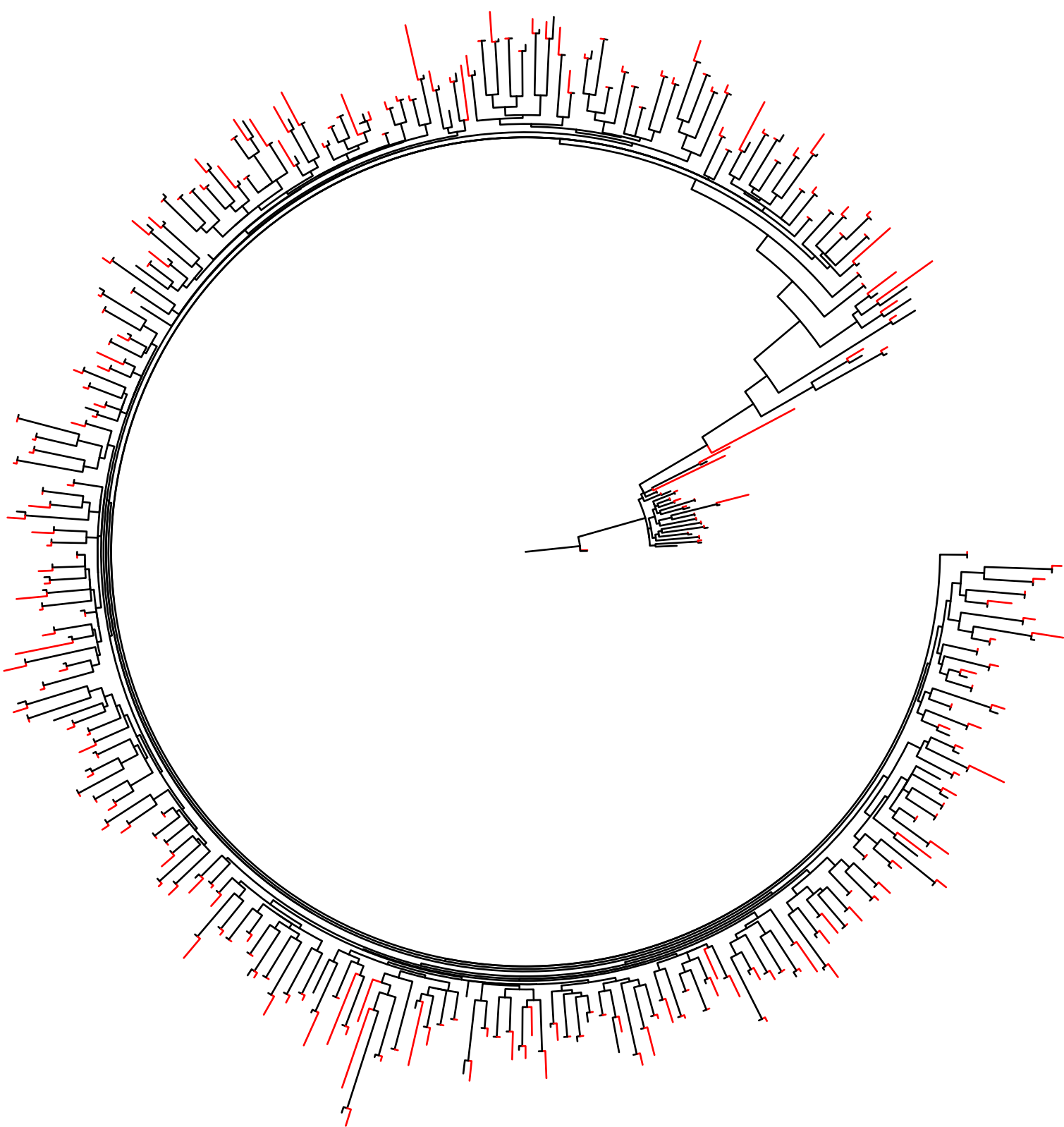

0.03

Supplement: Figure S1 — The maximum-likelihood phylogeny for the reverse transcriptase dataset. Foreground branches are marked in red. All terminal foreground branches lead to sequences obtained from patients who had been receiving antiretroviral therapy. (PDF) [file pcbi.1002507.s001.pdf]

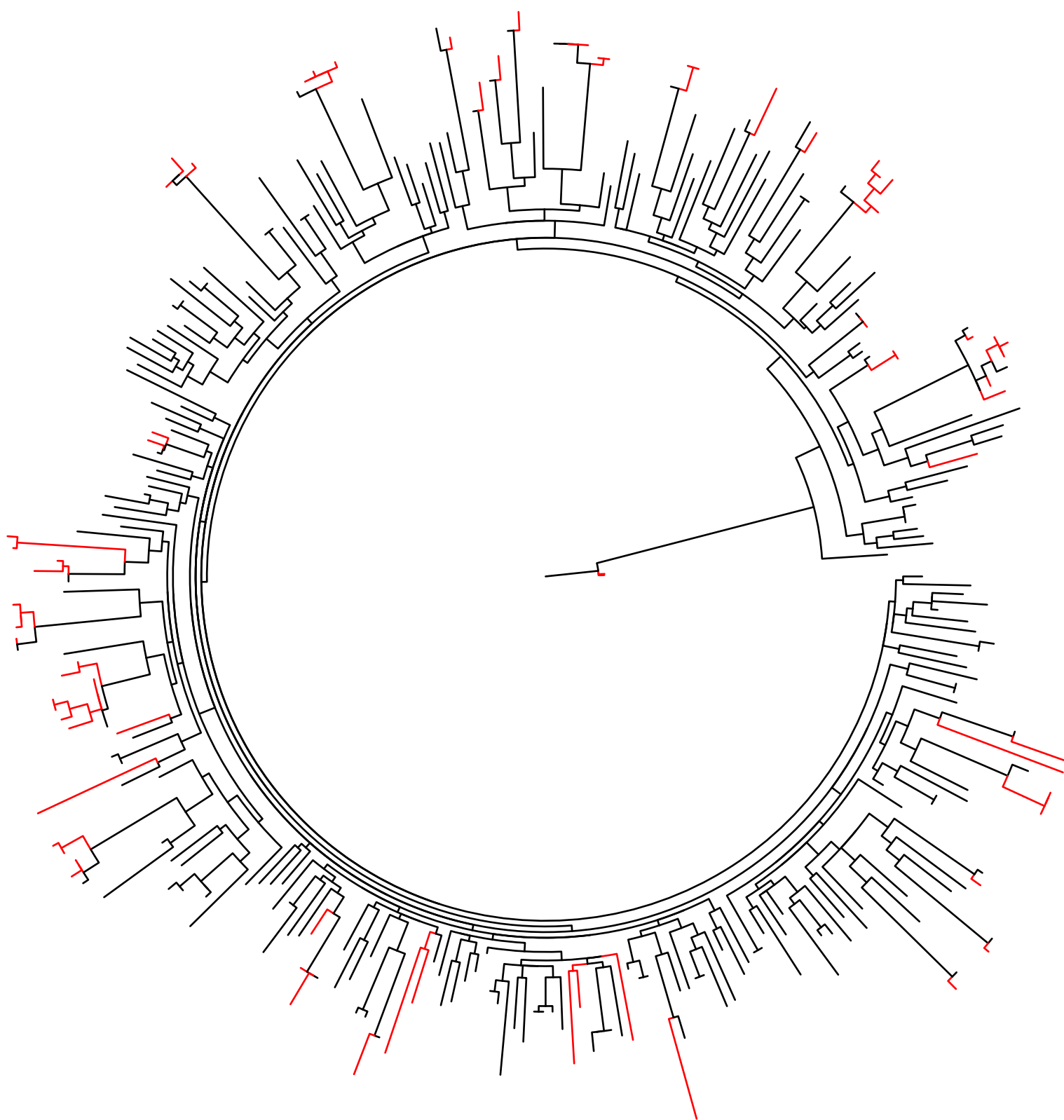

0.02

Supplement: Figure S2 — The maximum-likelihood phylogeny for the integrase dataset. Foreground branches are marked in red. All terminal foreground branches lead to sequences obtained from patients who had been receiving antiretroviral therapy. (PDF) [file pcbi.1002507.s002.pdf]

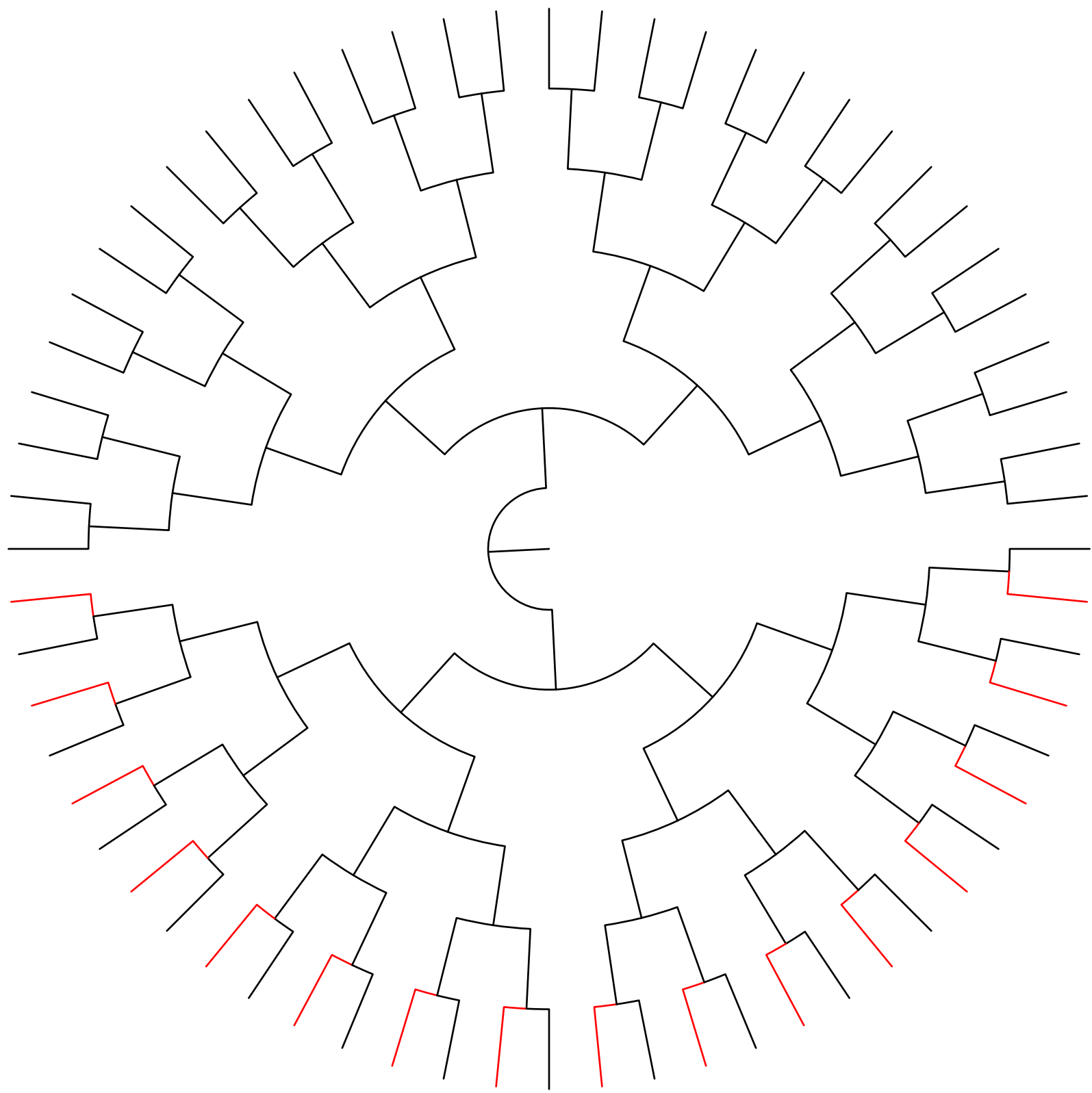

0.04

Supplement: Figure S3 — A balanced phylogeny used for simulations. Foreground branches are marked in red. See Text S1 for further simulation details. (PDF) [file pcbi.1002507.s003.pdf]
