# Supplementary material for: Modeling HIV-1 Drug Resistance as Episodic Directional Selection
Source: PLoS Comput Biol. 2012 May 10;8(5):e1002507. doi: 10.1371/journal.pcbi.1002507 (PMC3349733; doi:10.1371/journal.pcbi.1002507)
Supplement: Table S1 — Reverse transcriptase results - DEPS. (PDF) [file pcbi.1002507.s004.pdf]

Reverse transcriptase results - DEPS

Site	AA	Bayes factor	Resistance
39	T	3610.3	
48	T	3558.6	
49	R	5715.3	
60	I	416.3	
69	D	223.3	NRTI accessory
83	K	211.7	
98	S	$> 10^5$	
100	I	148.2	NNRTI Major
102	Q	153.2	
103	N	$> 10^5$	NNRTI Major
116	Y	317.9	NRTI
121	H	$> 10^5$	
121	Y	$> 10^5$	
122	E	3991	
122	P	$> 10^5$	
135	T	$> 10^5$	NNRTI accessory
142	T	4078.6	
158	S	3316.8	
162	C	$> 10^5$	
165	I	852	
166	R	1036	
169	D	$> 10^5$	
171	Y	665.7	
178	M	386.4	
179	D	116.5	NNRTI
181	C	102.5	NNRTI Major
184	V	$> 10^5$	NRTI Major
188	L	$> 10^5$	NNRTI Major
195	L	126.6	
196	E	$> 10^5$	
207	E	444.9	
207	Q	2162.6	
211	K	1167.1	
214	L	$> 10^5$	
230	L	374.2	NNRTI Major
243	H	271	
245	E	$> 10^5$	
245	M	490.4	
245	Q	$> 10^5$	
272	A	$> 10^5$	
272	P	$> 10^5$	
277	R	2872.6	
286	A	$> 10^5$	
288	S	$> 10^5$	
296	S	520.3	
297	K	132.3	
