# Supplementary material for: Modeling HIV-1 Drug Resistance as Episodic Directional Selection
Source: PLoS Comput Biol. 2012 May 10;8(5):e1002507. doi: 10.1371/journal.pcbi.1002507 (PMC3349733; doi:10.1371/journal.pcbi.1002507)
Supplement: Table S2 — Protease results - DEPS. (PDF) [file pcbi.1002507.s005.pdf]

Protease results - DEPS

Site	AA	Bayes factor	Resistance
12	T	$> 10^5$	
60	E	3677.6	PI Accessory
61	E	$> 10^5$	
90	M	$> 10^5$	PI Major
