# Supplementary material for: Modeling HIV-1 Drug Resistance as Episodic Directional Selection
Source: PLoS Comput Biol. 2012 May 10;8(5):e1002507. doi: 10.1371/journal.pcbi.1002507 (PMC3349733; doi:10.1371/journal.pcbi.1002507)
Supplement: Table S3 — Integrase results - DEPS. (PDF) [file pcbi.1002507.s006.pdf]

Integrase results - DEPS

Site	AA	Bayes factor	Resistance
10	D	$> 10^5$	
11	D	$> 10^5$	
14	R	1962.8	
17	N	$> 10^5$	
23	V	101.1	
31	I	498.3	
39	C	$> 10^5$	
45	V	$> 10^5$	
74	M	810.3	RAL Accessory
97	A	161.9	RAL Accessory
101	I	$> 10^5$	
111	T	$> 10^5$	
112	V	155.8	
113	V	265.7	
119	G	145.6	
119	P	$> 10^5$	
122	I	$> 10^5$	
124	A	$> 10^5$	
124	N	$> 10^5$	
125	A	$> 10^5$	
126	L	137	
140	S	$> 10^5$	INI Major
143	R	$> 10^5$	RAL Major
148	H	924	INI Major
155	H	$> 10^5$	INI Major
156	N	$> 10^5$	
193	E	2608.3	
201	I	$> 10^5$	INI other
203	M	$> 10^5$	RAL accessory
206	S	$> 10^5$	
208	L	793.9	
215	N	160.3	
216	H	229	
220	L	961.8	
227	F	$> 10^5$	
230	N	$> 10^5$	RAL accessory
256	E	$> 10^5$	
265	V	$> 10^5$	
283	G	3559.4	
