# Supplementary material for: Modeling HIV-1 Drug Resistance as Episodic Directional Selection
Source: PLoS Comput Biol. 2012 May 10;8(5):e1002507. doi: 10.1371/journal.pcbi.1002507 (PMC3349733; doi:10.1371/journal.pcbi.1002507)
Supplement: Table S4 — Reverse Transcriptase - MEDS: Maximum likelihood parameter values for the test for episodic directional selection. (PDF) [file pcbi.1002507.s007.pdf]

Reverse Transcriptase - MEDS: Maximum likelihood parameter values for the test for episodic directional selection

Site	AA	L_{alt}	p	ω_T	β^F	β^B	α	L_{null}	β^F_{null}	β^B_{null}	α_{null}
41	L	-51.926	0.000129449	1936.984496	0.00251767	0.0421996	3.62273	-59.2509	0.42699	0.0421975	3.55972
64	K	-73.5638	0.000122162	11.9947761	28.0258	0.163148	0.144539	-80.9434	1.2216	0.180671	0.144415
98	S	-158.942	0.000244148	999999	6.92E-06	0.499682	0.351024	-165.67	0.395184	0.519358	0.351024
100	I	-148.112	1.14E-08	2576.319588	0.00950148	0	1.56231	-164.402	3.27566	0	1.56068
103	N	-279.906	0	629.9148265	0.42715	0.159333	1.45947	-467.417	36.6318	0.159454	1.46635
104	Y	-105.077	0.000121947	199999	0.26106	0.158995	2.05124	-112.458	0.54364	0.158985	2.04216
116	Y	-99.5475	0.000159688	1287.659794	0.00537767	0	4.35354	-106.675	0.646434	0	4.35297
151	M	-94.5339	9.65E-11	199999	1.37238	0	0.931292	-115.479	2.67459	0	0.928198
151	Q	-105.882	1.18E-05	13.96804323	59.045	0	0.911675	-115.479	2.67459	0	0.928198
165	L	-94.7785	2.48E-06	166665.6667	0.00075301	0.456091	0.085129	-105.87	0.46651	0.444944	0.0850503
181	I	-128.417	3.15E-10	2221.222222	3.21155	0.0439401	3.31344	-148.207	4.42602	0.0437376	3.31536
184	V	-155.372	0	25.82331483	1.35934	0.0415552	4.39214	-203.578	8.42545	0.0416161	3.35E-14
188	L	-53.051	5.18E-08	377.9314134	1.80625	0	0.17763	-67.8746	3.00599	0	0.177534
188	Y	-56.0418	1.15E-06	17.61330852	574.455	0	1.32E-15	-67.8746	3.00599	0	0.177534
190	S	-159.906	4.44E-13	75.85213649	1.67995	0.0290578	1.51613	-186.126	3.4167	0.0290576	1.515
215	F	-126.15	0.000141025	160.6553508	1.08574	0.185441	0.437698	-133.394	1.50103	0.185461	0.437692
215	T	-124.192	1.79E-05	15.18961274	24.8512	0.185635	0.437508	-133.394	1.50103	0.185461	0.437692
228	R	-141.874	1.46E-05	72.20644217	0.276463	0.0665712	1.5965	-151.271	1.29717	0.0664674	1.53164
230	L	-20.3123	0.00014829	308.9814011	0.0152005	0	3.52E-14	-27.5092	0.427747	0	9.96E-15
286	A	-101.909	4.23E-05	20832.33333	0.00123901	1.42472	0.366754	-110.292	2.85736	1.53698	0.366759
