# Supplementary material for: Modeling HIV-1 Drug Resistance as Episodic Directional Selection
Source: PLoS Comput Biol. 2012 May 10;8(5):e1002507. doi: 10.1371/journal.pcbi.1002507 (PMC3349733; doi:10.1371/journal.pcbi.1002507)
Supplement: Table S5 — Reverse Transcriptase - FEEDS: Maximum likelihood parameter values for the test for episodic diversifying selection. (PDF) [file pcbi.1002507.s008.pdf]

Reverse Transcriptase - FEEDS: Maximum likelihood parameter values for the test for episodic diversifying selection

Site	L_{alt}	p	β^F	β^B	α	L_{null}	β^B_{null}	α_{null}
64	-80.9434	0.00673333	1.2216	0.180671	0.144415	-84.6147	0.192483	0.293385
102	-57.0195	0.00246648	0.707931	0.21164	0	-61.6022	0.23243	0.0877115
103	-467.417	0	36.6318	0.159454	1.46635	-522.478	0.15939	19.2034
188	-67.8746	0.000223477	3.00599	0	0.177534	-74.6859	0	1.24507
200	-304.137	1.68E-05	2.99932	1.81408	0.250523	-313.398	1.90785	0.955633
245	-400.305	0.000555886	2.4642	3.01382	0.355555	-406.264	3.09518	0.6565
