# Supplementary material for: Modeling HIV-1 Drug Resistance as Episodic Directional Selection
Source: PLoS Comput Biol. 2012 May 10;8(5):e1002507. doi: 10.1371/journal.pcbi.1002507 (PMC3349733; doi:10.1371/journal.pcbi.1002507)
Supplement: Table S6 — Protease - MEDS: Maximum likelihood parameter values for the test for episodic directional selection. (PDF) [file pcbi.1002507.s009.pdf]

Protease - MEDS: Maximum likelihood parameter values for the test for episodic directional selection

Site	AA	L_{alt}	p	ω_T	β^F	β^B	α	L_{null}	β^F_{null}	β^B_{null}	α_{null}
12	T	-103.612	3.65E-08	28.87839493	1.09748	1.3625	0.635411	-118.775	2.29036	1.35548	0.63554
13	V	-24.8011	0.000295628	490.1591356	0.00930203	0.139647	1.00E-06	-31.3501	1.60248	0.139706	0
35	D	-88.9355	0.000173524	8.556666253	1.90179	0.871693	1.32226	-95.9845	1.69196	0.886322	1.31565
60	E	-73.035	1.35E-06	7574.757576	0.00156274	0.695636	2.69379	-84.7105	1.24539	0.696732	2.9248
61	E	-52.3855	1.28E-06	10868.56522	0.00172908	0.583621	1.65483	-64.111	1.82882	0.583915	1.65465
74	S	-73.7552	3.30E-05	19.92882108	1.56302	0.651228	0	-82.375	2.30865	0.654976	0
84	V	-29.3416	0.000398906	890.2655971	0.00491864	0.144372	0.812221	-35.6102	1.58973	0.149308	0.80007
90	M	-30.7741	0	1903.761905	0.050944	0.308249	1.79E-16	-79.3434	7.57007	0.309395	0
93	L	-10.3112	0.000388636	249999	7.48786	0.17605	2.85E-16	-16.6042	0.303064	0.176431	0
