# Supplementary material for: Modeling HIV-1 Drug Resistance as Episodic Directional Selection
Source: PLoS Comput Biol. 2012 May 10;8(5):e1002507. doi: 10.1371/journal.pcbi.1002507 (PMC3349733; doi:10.1371/journal.pcbi.1002507)
Supplement: Table S7 — Protease - FEEDS: Maximum likelihood parameter values for the test for episodic diversifying selection. (PDF) [file pcbi.1002507.s010.pdf]

Protease - FEEDS: Maximum likelihood parameter values for the test for episodic diversifying selection

Site	L_{alt}	p	β^F	β^B	α	L_{null}	β^B_{null}	α_{null}
10	-81.675	0.000458655	5.8636	0.543275	0.664002	-87.8134	0.54534	3.00957
54	-72.6876	0.00256859	4.34929	0.169313	0.965253	-77.2331	0.174386	1.96659
71	-39.1825	0.00107298	2.43536	0.12767	5.74E-16	-44.5311	0.127734	1.30452
74	-82.375	0.00134268	2.30865	0.654976	0	-87.5164	0.656849	1.28352
82	-58.5682	2.61E-05	3.6972	0.3434	0.106117	-67.4086	0.344812	1.1085
90	-79.3434	7.54E-11	7.57007	0.309395	0	-100.53	0.33163	1.56387
