# Supplementary material for: Modeling HIV-1 Drug Resistance as Episodic Directional Selection
Source: PLoS Comput Biol. 2012 May 10;8(5):e1002507. doi: 10.1371/journal.pcbi.1002507 (PMC3349733; doi:10.1371/journal.pcbi.1002507)
Supplement: Table S8 — Integrase - MEDS: Maximum likelihood parameter values for the test for episodic directional selection. (PDF) [file pcbi.1002507.s011.pdf]

Integrase - MEDS: Maximum likelihood parameter values for the test for episodic directional selection

Site	AA	L_{alt}	p	ω_T	β^F	β^B	α	L_{null}	β^F_{null}	β^B_{null}	α_{null}
72	I	-179.618	0.000475054	2679.965147	0.00551947	2.85548	0.542935	-185.724	1.62786	2.88008	0.5433
97	A	-26.1408	0.000137831	336.9520108	0.0279966	0	1.00E-06	-33.4066	3.31582	0	0
140	S	-126.451	3.00E-08	5375.344086	0.00410666	0.0318389	1.62144	-141.806	6.97925	0.0514961	1.62096
143	R	-49.3462	7.27E-05	23.50740124	8.21593	0	1.00E-06	-57.2152	11.5732	0	0
148	H	-92.8819	4.72E-10	35.4976824	1.9819	0.309085	1.07857	-112.276	9.8634	0.301922	1.08205
155	H	-57.8204	3.33E-16	5713.285714	0.0178668	0	1.13413	-91.1401	7.44601	0	1.09009
