# Supplementary material for: Modeling HIV-1 Drug Resistance as Episodic Directional Selection
Source: PLoS Comput Biol. 2012 May 10;8(5):e1002507. doi: 10.1371/journal.pcbi.1002507 (PMC3349733; doi:10.1371/journal.pcbi.1002507)
Supplement: Table S9 — Integrase - FEEDS: Maximum likelihood parameter values for the test for episodic diversifying selection. (PDF) [file pcbi.1002507.s012.pdf]

Integrase - FEEDS: Maximum likelihood parameter values for the test for episodic diversifying selection

Site	L_{alt}	p	β^F	β^B	α	L_{null}	β^B_{null}	α_{null}
97	-33.4066	2.17E-05	3.31582	0	0	-42.4215	0	0.901595
140	-141.806	0.000279464	6.97925	0.0514961	1.62096	-148.407	0.0536062	2.34193
143	-57.2152	3.48E-13	11.5732	0	0	-83.6744	0	1.62752
148	-112.276	4.22E-05	9.8634	0.301922	1.08205	-120.661	0.308504	3.56771
155	-91.1401	0.000646024	7.44601	0	1.09009	-96.9593	0	3.10661
227	-40.3814	0.00640165	1.48678	0.230169	0	-44.0982	0.230209	0.229592
230	-95.5463	0.00482776	1.13302	0.632964	0	-99.5177	0.632944	0.299417
