# Supplementary material for: Modeling HIV-1 Drug Resistance as Episodic Directional Selection
Source: PLoS Comput Biol. 2012 May 10;8(5):e1002507. doi: 10.1371/journal.pcbi.1002507 (PMC3349733; doi:10.1371/journal.pcbi.1002507)
Supplement: Text S1 — Simulation details. The variation in nuisance parameters used for our simulations. (PDF) [file pcbi.1002507.s013.pdf]

Simulation details. We ran 4 simulations for MEDS. We used equal nucleotide substitution rates ($\theta_{ij} = 1$), and equal target nucleotide frequency parameters ($\pi_{ij} = 0.25$). The t parameter (as in e^{Qt}) for every branch was set to 0.05. We also needed to choose the synonymous rate α, the background non-synonymous rate β^B, and the foreground non-synonymous rate β^F. We let each of these be either 0.2 or 1, simulating sites with all possible combinations of these parameters. For example, if $\alpha = 0.2$, $\beta^B = 0.2$ but $\beta^F = 1$, we would have neutral selection on the background and diversifying selection on the foreground. ω_T assumed one of 5 values (2, 5, 10, 100, 1000), also described in the main text. An example of a balanced phylogenies used, along with the location of the foreground branches, is shown in Figure S3.
